# Osteomyelitis due to *Clostridium innocuum* in a patient with acute lymphoblastic leukemia: case report and literature review

**DOI:** 10.1186/s40064-015-1176-3

**Published:** 2015-07-29

**Authors:** Yoshikazu Mutoh, Risen Hirai, Akira Tanimura, Takashi Matono, Eriko Morino, Satoshi Kutsuna, Maki Nagamatsu, Norio Ohmagari, Shotaro Hagiwara

**Affiliations:** Division of Hematology, Department of Internal Medicine, National Center for Global Health and Medicine, 1-21-1 Toyama, Shinjuku, Tokyo, 162-8655 Japan; Disease Control and Prevention Center, National Center for Global Health and Medicine, 1-21-1 Toyama, Shinjuku, Tokyo, 162-8655 Japan; Department of Respiratory Diseases, National Center for Global Health and Medicine, Tokyo, Japan; Department of Infectious Diseases, Research Institute, National Center for Global Health and Medicine, Tokyo, Japan

**Keywords:** *Clostridium innocuum*, Acute lymphoblastic leukemia, Osteomyelitis, Direct 16S-rRNA sequencing, Neutropenic enterocolitis

## Abstract

**Introduction:**

*Clostridium innocuum* is an anaerobic Gram-positive bacterium, unable to produce toxins and rarely causes infections. We report the first case of *C. innocuum* osteomyelitis and bacteremia in a patient with acute lymphoblastic leukemia (ALL). Findings were compared with previously reported cases of *C. innocuum* infections in immunocompromised patients, e.g., patients with acquired immune deficiency syndrome, leukemia, and organ transplantation.

**Case description:**

A 32-year-old Japanese male was admitted for persistent low-grade fever and purpura lasting for 1 month. Complete blood counts and cytogenetic analysis identified Ph1-positive ALL, which was successfully treated using chemotherapy. However, the patient developed high fever and lumbar pain during complete remission. Fluorodeoxyglucose-positron emission tomography and computed tomography demonstrated osteomyelitis. *C. innocuum* was identified as the causative agent and the patient was successfully treated using antibiotic therapy.

**Discussion and evaluation:**

We performed a literature review revealing a number of common aspects to the clinical presentation of *C. innocuum* infection and an association with various comorbidities. Further, we highlight the most efficient diagnostic and treatment strategies for *C. innocuum* osteomyelitis.

**Conclusions:**

*Clostridium innocuum* can be a causative pathogen of osteomyelitis and bacteremia in immunocompromised patients.

**Electronic supplementary material:**

The online version of this article (doi:10.1186/s40064-015-1176-3) contains supplementary material, which is available to authorized users.

## Introduction

Clostridial species are common anaerobic, spore-forming, Gram-positive bacteria found in the normal flora of the oropharynx and gastrointestinal tract. Among them, *Clostridium innocuum* is an unusual cause of infections in humans. A few reports have described bacteremia due to *C. innocuum* in immunocompromised patients, such as those with acquired immune deficiency syndrome (AIDS), leukemia, and organ transplantation. Because *C. innocuum* has intrinsic resistance to several common antibiotics, including vancomycin, it may cause intractable infections (Alexander et al. 1995; David et al. 2004). We report the first case of pelvic osteomyelitis and sepsis due to *C. innocuum* infection in a patient with acute lymphocytic leukemia (ALL). We performed a literature review of previous reports to determine the most appropriate diagnostic strategies and treatment regimens in cases of *C. innocuum* infection in patients with distinct comorbidities.

## Review

### Case description

A 32-year-old Japanese male with no previous medical history was admitted to our hospital for a persistent low-grade fever and purpura lasting for 1 month. A complete blood count (CBC) revealed marked anemia, thrombocytopenia, and hyperleukocytosis (136,130/µL; blasts 97%, neutrophils 0%, lymphocytes 2%, and eosinophils 1%). Bone marrow aspiration from iliac crest were hypercellular with 97.6% lymphoblasts. Cytogenetic analysis revealed that blasts were positive for the Philadelphia chromosome (Ph1) with minor BCR/ABL mRNA transcripts. Therefore, the patient was diagnosed with Ph1-positive ALL.

**Fig. 1:**
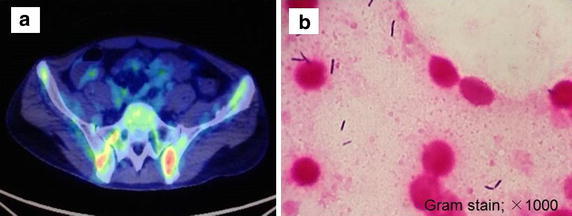
Diagnostic approach for a 32-year-old male with *Clostridium innocuum* osteomyelitis. **a** (18F)-Fluorodeoxyglucose positron emission tomography (FDG-PET) revealed marked uptake of FDG in the sacroiliac joint and iliac bone. **b** Bone marrow biopsy from iliac crest confirmed *C. innocuum* infection (Grambiopsy conf1000).

Combination chemotherapy with dasatinib was immediately initiated as remission induction. On treatment day 27, CBCs had normalized and bone marrow aspiration from iliac crest analysis confirmed complete remission. On treatment day 39, consolidation chemotherapy with daunorubicin, cyclophosphamide, vincristine, prednisolone, methotrexate, and dasatinib was initiated. On treatment day 51, the patient became pyrexial (39.2°C) and reported severe lower back pain. The pain rapidly worsened and radiated to the right axilla. WBC count was 430/mm^3^, with 8% segmented neutrophils, 2% bands, 80% lymphocytes, and 10% monocytes. We immediately collected blood culture and started empiric antibiotic therapy with meropenem, vancomycin, and liposomal amphotericin B. CT imaging revealed no apparent abnormal findings; however, fluorodeoxyglucose (18F)-positron emission tomography (FDG-PET) revealed increased uptake of FDG in the iliac bones and right side of the sacrum. These findings suggested osteomyelitis of the iliac bone and sacrum and Gram-positive bacteria were detected by needle aspiration biopsy of the iliac bone (Fig. 1). Gram-positive bacterium was also found in the blood culture; however, the bacteria could not be identified. For this reason, we tested the blood culture for direct 16S ribosomal RNA (16S rRNA) sequencing and identified *C. innocuum* 2 days later. The presence of this pathogen was also confirmed by bone marrow culture from iliac crest. As a result of these findings, the patient was treated with piperacillin/tazobactam, metronidazole, and clindamycin. In this case, the causative pathogen was sensitive to ampicillin, piperacillin/tazobactam, meropenem, clindamycin, and metronidazole. Intermediate sensitivity to penicillin G and cefmetazole was observed. Further, the isolated strain was resistant to vancomycin with a minimum inhibitory concentration (MIC) of 8 μg/mL.

Fever gradually resolved over the next 3 weeks, but the lumbar pain persisted. CT imaging identified a small abscess in the iliacus muscle. Therefore, CT-guided drainage was performed. No pathogens were detected in cultures of the abscess fluid or blood. The previously administered antibiotic regimen was consequently deemed effective. After another 8 weeks of antibiotic therapy, the lumbar pain subsided and treatment was terminated. On treatment day 104, the patient was asymptomatic and chemotherapy was reinitiated.

## Discussion and evaluation

We identified previously published cases of *C. innocuum* infection by conducting a PubMed search of the literature using the following keywords: *Clostridium innocuum*; ALL; osteomyelitis; and anaerobic bacteria. PubMed queries also included infections that developed in immunocompromised patients during chemotherapy.

The first human case of *C. innocuum* infection was reported in the 1960s (Smith and King 1962). The term “innocuum” is derived from “innocuous” (i.e., meaning innocent) as the organism lacks the ability to produce toxins. A PubMed search of the literature identified 16 previously reported cases of *C. innocuum* infection (Smith and King 1962; Castiglioni et al. 2003; Crum-Cianflone 2009; Hung et al. 2014; Bodey et al. 1991; Cutrona et al.^1995^). Details of these cases are summarized in Additional file 1: Table S1. Median age of patients was 38.0 years, and 66.7% were male. All but one patient had a comorbid disorder, namely acute leukemia, AIDS, chronic hepatitis, genitourinary malignancy, gastro-intestinal malignancy, or organ transplantation (Crum-Cianflone 2009; Hung et al. 2014; Bodey et al. 1991; Cutrona et al. 1995; Shah et al. 2009). The most common clinical symptom was fever of unknown origin followed by the gastrointestinal tract disorder, such as diarrhea or constipation, and/or respiratory disorder. Almost all patients developed bacteremia. Most commonly used agents were piperacillin/tazobactam, metronidazole, and clindamycin to which *C. innocuum* appeared susceptible. Nevertheless, the prognosis of these patients was poor, with a mortality rate of 33.3%.

The diagnosis of anaerobic infections is clinically challenging. Detection rates from blood cultures are extremely low (approximately 4.0%), mainly because of the slow growth of anaerobic bacteria. In addition, a number of anaerobic bacteria species are part of the normal microbiota in humans and concomitant infection with aerobic bacteria is common (Cockerill et al. 1997). One study in patients with blood cultures positive for anaerobic bacteria demonstrated patients who received appropriate anti-anaerobic antibiotics as the initial treatment, or who were immediately changed to appropriate antibiotics following identification of the causative pathogen, had markedly improved prognosis compared with patients who did not receive appropriate antibiotic treatment (Salonen et al. 1998).

Direct 16S ribosomal RNA gene sequencing has recently been developed, allowing the composition of microbial communities to be analyzed. In the present case, this approach identified *C. innocuum* in blood cultures within a few days of admission and that allowed the selection of an appropriate initial antibiotic regimen. Therefore, direct 16S ribosomal RNA gene sequencing may be useful in the early detection of Clostridium species in clinical samples (Drancourt et al. 2000; Mory et al. 1998). Unlike *C. difficile*, *C. innocuum* is susceptible to penicillin, clindamycin, and metronidazole but not to vancomycin (Ackermann et al. 2001; Goldstein et al. 2014). Our patient was successfully treated by a 8-week combination antibiotic therapy comprising piperacillin/tazobactam, clindamycin, and metronidazole. There is currently no standard duration of treatment for *C. innocuum* infections. However, 4–6 weeks with appropriate antibiotics and debridement is generally recommended for the treatment of osteomyelitis (Howard et al. 1994; Spellberg and Lipsky 2012).

Comprehensive blood and bone marrow aspiration analysis revealed Ph1-positive ALL in our patient warranting immediate initiation of systemic chemotherapy. The patient developed high fever and back pain, although complete remission of ALL was achieved. Therefore, we suspected that these symptoms were due to an infection rather than related to a lymphoproliferative disease.

Systemic chemotherapy has been reported to induce neutropenic enterocolitis (NEC), a common complication in neutropenic cancer patients (Nesher and Rolston 2013). Symptoms generally develop after the third week of chemotherapy and include neutropenic fever and abdominal pain (mainly in the right lower abdomen). The diagnostic criteria for NEC is neutropenia (absolute neutrophil count <500 × 10^6^ cells/L), bowel wall thickening >4 mm, and high fever with the exclusion of other diagnoses. The mortality of NEC patients is relatively high (>60%). Risk factors for NEC include acute leukemia, lymphoma, solid tumor, and neutropenia in addition to cytotoxic chemotherapeutic agents such as cytosine arabinoside, anthracyclines, and taxanes. In this case, the patient complained of abdominal distention and constipation after consolidation chemotherapy was started. Computed tomography (CT) revealed marked constipation and edematous intestinal wall, findings that were compatible with NEC. Therefore, we believed that bacterial translocation of *C. innocuum* occurred from the damaged intestinal tract to the iliac bone.

According to identified case reports of *C. innocuum*, previous *C. difficile* associated diarrhea (CDAD) may predispose to *C. innocuum* bacteremia (Crum-Cianflone 2009); however, *C. difficile* infection was not detected in our case. NEC without CDAD may induce bacterial translocation of *C. innocuum* from the enteral flora. Osteomyelitis due to Clostridium species is quite rare. In the previously reported articles on osteomyelitis due to *C. difficile*, *C. clostridioforme*, *C. celerecrescens*, *C. bifermentans*, and *C. septicum*, several have reported an association with trauma (Mischnik et al. 2011; Al-Najjar et al. 2013; Scanlan et al. 1994). The treatment that was most commonly chosen was metronidazole, clindamycin, or β-lactam. Almost all anaerobic osteomyelitis occur by direct extension from an adjacent focus of infection and are rarely due to bacteremia; however, in the present case, the bacteria may have reached from the intestinal tract to the iliac bone via blood circulation.

Immunodeficiency may contribute to the progression of infection. As pathogens are difficult to identify, treatments generally include broad spectrum antibiotics covering rare anaerobic bacteria and antifungal agents. Surgical intervention is recommended only in cases of bowel perforation or necrosis. The recommended duration of antibiotic therapy is from 4 weeks to 6 months. For immunosuppressed patients, the recommended treatment is more than 8 weeks. Further, it is considered that less than 4 weeks of antibiotic therapy is a risk factor for recurrence in patients who have undergone surgical management (Pigrau et al. 2015; Lima et al. 2014).

## Conclusions

In conclusion, we report a rare case of osteomyelitis and bacteremia due to *C. innocuum.* Although *C innocuum* lacks the ability to produce toxins and has weak pathogenicity, it may cause severe infections in immunocompromised patients, such as those with acute leukemia, chronic viral hepatitis, or HIV.

## Consent

Informed consent for publication of this report and any accompanying images was obtained from the patient.

## Additional file

Additional file 1: Table S1. Reported cases of *C. innocuum* infection
