# Supplementary material for: Osteomyelitis due to Clostridium innocuum in a patient with acute lymphoblastic leukemia: case report and literature review
Source: Springerplus. 2015 Jul 29;4:385. doi: 10.1186/s40064-015-1176-3 (PMC4518021; doi:10.1186/s40064-015-1176-3)
Supplement: Additional file 1: Table S1. — Reported cases of C. innocuum infection [file 40064_2015_1176_MOESM1_ESM.docx]

| No | Ref | Age | Sex | Comorbid illness | Clinical symptom | infection | Treatment | Outcome |
| --- | --- | --- | --- | --- | --- | --- | --- | --- |
| 1 | our case | 32 | M | leukemia | Back pain, Fever | osteomyelitis | piperacillin/tazobactam, clindamycin,  metronidazole | Survived |
| 2 | 2014　[6] | 85 | M | CDAD※  Cytomegalovirus colitis | Diarrhea | bacteremia | piperacillin/tazobactam | Survived |
| 3 | 2009　[5] | 38 | M | AIDS | Fever | bacteremia | Metronidazole, daptomycin,  linezolid | Survived |
| 4 | 2003　[4] | 38 | F | chronic hepatitis C  kidney transplantation | Abdominal discomfort, Fever | bacteremia | penicillin G, clindamycin,  piperacillin/tazobactam | Survived |
| 5 | 1995　[8] | 18 | F | psychiatric disorder | SOB, Headache, Productive cough | endocarditis | erythromycin, ceftriaxone | Died |
| 6 | 1991　[7] | 31 | M | melanoma | Fever | bacteremia | unknown | Survived |
| 7 | 1991　[7] | 70 | M | undifferentiated malignancy | Fever, Abdominal pain | bacteremia | unknown | Survived |
| 8 | 1991　[7] | 39 | F | leukemia | Fever | bacteremia | unknown | Survived |
| 9 | 1991　[7] | 28 | F | leukemia | Fever | bacteremia | unknown | Survived |
| 10 | 1991　[7] | 36 | M | Lymphoma | Catheter-associated soft tissue infection | bacteremia | unknown | Survived |
| 11 | 1991　[7] | 65 | M | leukemia | Fever | bacteremia | unknown | Died |
| 12 | 1991　[7] | 54 | M | leukemia | Fever | bacteremia | unknown | Died |
| 13 | 1991　[7] | 49 | M | leukemia | Fever | bacteremia | unknown | Survived |
| 14 | 1991　[7] | 27 | F | leukemia | Ileus, multiple colonic submucosal hemorrhages | bacteremia | unknown | Died |
| 15 | 1991　[7] | 59 | M | leukemia | Fever | bacteremia | unknown | Died |
| 16 | 2009　[9] | unknown | unknown | prostate cancer | unknown | bacteremia | unknown | Died |
| 17 | 2009　[9] | unknown | unknown | renal cell carcinoma | unknown | bacteremia | unknown | Died |

※CDAD: Clostridium difficile-associated diarrhea

Table: Reported cases of *C.innocuum* infection.
